# Targeting Hypoxia-Inducible Factor-1 (HIF-1) in Cancer: Emerging Therapeutic Strategies and Pathway Regulation

**DOI:** 10.3390/ph17020195

**Published:** 2024-02-01

**Authors:** Reem A. Qannita, Ayah I. Alalami, Amani A. Harb, Shereen M. Aleidi, Jalal Taneera, Eman Abu-Gharbieh, Waseem El-Huneidi, Mohamed A. Saleh, Karem H. Alzoubi, Mohammad H. Semreen, Mohammad Hudaib, Yasser Bustanji

**Affiliations:** 1 Research Institute of Medical and Health Sciences, University of Sharjah, Sharjah 27272, United Arab Emirates; u22103857@sharjah.ac.ae (R.A.Q.); u22103859@sharjah.ac.ae (A.I.A.); jtaneera@sharjah.ac.ae (J.T.); eabugharbieh@sharjah.ac.ae (E.A.-G.); welhuneidi@sharjah.ac.ae (W.E.-H.); mohamed.saleh@sharjah.ac.ae (M.A.S.); kelzubi@sharjah.ac.ae (K.H.A.); msemreen@sharjah.ac.ae (M.H.S.); 2 College of Medicine, University of Sharjah, Sharjah 27272, United Arab Emirates; 3 Department of Basic Sciences, Faculty of Arts and Sciences, Al-Ahliyya Amman University, Amman 19111, Jordan; a.harb@ammanu.edu.jo; 4 School of Pharmacy, The University of Jordan, Amman 11942, Jordan; s.aleidi@ju.edu.jo (S.M.A.); m.hudaib@ju.edu.jo (M.H.); 5 Department of Pharmacology and Toxicology, Faculty of Pharmacy, Mansoura University, Mansoura 35516, Egypt; 6 Department of Pharmacy Practice and Pharmacotherapeutics, College of Pharmacy, University of Sharjah, Sharjah 27272, United Arab Emirates; 7 Department of Medicinal Chemistry, College of Pharmacy, University of Sharjah, Sharjah 27272, United Arab Emirates

**Keywords:** hypoxia-inducible factor-1, hypoxia, cancer, HIF-1α, metabolism reprogramming, HIF-1 inhibitors, nanotechnology

## Abstract

Hypoxia-inducible factor-1 (HIF-1) is a key regulator for balancing oxygen in the cells. It is a transcription factor that regulates the expression of target genes involved in oxygen homeostasis in response to hypoxia. Recently, research has demonstrated the multiple roles of HIF-1 in the pathophysiology of various diseases, including cancer. It is a crucial mediator of the hypoxic response and regulator of oxygen metabolism, thus contributing to tumor development and progression. Studies showed that the expression of the HIF-1α subunit is significantly upregulated in cancer cells and promotes tumor survival by multiple mechanisms. In addition, HIF-1 has potential contributing roles in cancer progression, including cell division, survival, proliferation, angiogenesis, and metastasis. Moreover, HIF-1 has a role in regulating cellular metabolic pathways, particularly the anaerobic metabolism of glucose. Given its significant and potential roles in cancer development and progression, it has been an intriguing therapeutic target for cancer research. Several compounds targeting HIF-1-associated processes are now being used to treat different types of cancer. This review outlines emerging therapeutic strategies that target HIF-1 as well as the relevance and regulation of the HIF-1 pathways in cancer. Moreover, it addresses the employment of nanotechnology in developing these promising strategies.

## 1. Introduction

Normoxia, the normal physiological level of oxygenation, is a condition characterized by the exposure of cells or tissues to oxygen levels that are within the usual range. Although there is variation in oxygen levels across different organs, the typical range is often between 3 and 9%. Whereas, in vitro, the normal oxygen levels lie between 20 and 21% [1]. Under conditions of adequate oxygen availability, most cells engage in the production of adenosine triphosphate (ATP) through the process of oxidative phosphorylation. Hypoxia is a physiological state characterized by inadequate cellular oxygen levels, leading to an energy production shifting toward anaerobic metabolism. The presence of hypoxic conditions leads to a reduction in the rate of cell proliferation in normal (non-cancerous) cells, imposing a constraint on the expansion of new oxygen-consuming cells. The hypoxic environment is a defining feature of the tumor microenvironment, wherein the excessive and uncontrolled cellular division and proliferation lead to disparities in oxygen usage and an inadequate oxygen supply that exceeds the capacity of the local vascular system [2]. Cancer cells can adapt to hypoxic conditions by modulating their metabolic pathways through changes in gene expression and enzyme activity [3]. Hypoxia-inducible factors (HIFs) play a critical role in the regulating this adaptive process and controlling the expression of genes associated with cancer development and resistance to treatment [4,5]. Recent research has revealed that in the context of hypoxia within tumor cells, HIFs play a crucial role in orchestrating the metabolic changes observed in various substances such as glucose, lactate, lipids, and amino acids. Thus, the significance of HIFs in the development of tumors and the advancement of tumor growth is underscored [6,7].

The targeting of HIFs has been recognized as a prospective strategy for enhancing cancer therapies [8,9,10]. A number of small molecular inhibitors targeting HIF have been developed and incorporated into various drug delivery systems. However, unfortunately, only a limited subset of these inhibitors is currently being evaluated in clinical trials.

The current review provides a comprehensive summary of the latest research discoveries pertaining to the molecular pathways of tumor-associated HIFs, the advancements in developing HIF inhibitors, and the possible utilization of nanomaterials as techniques for cancer treatment.

## 2. Structure and Functions of HIF Isoforms

**Figure 1:**
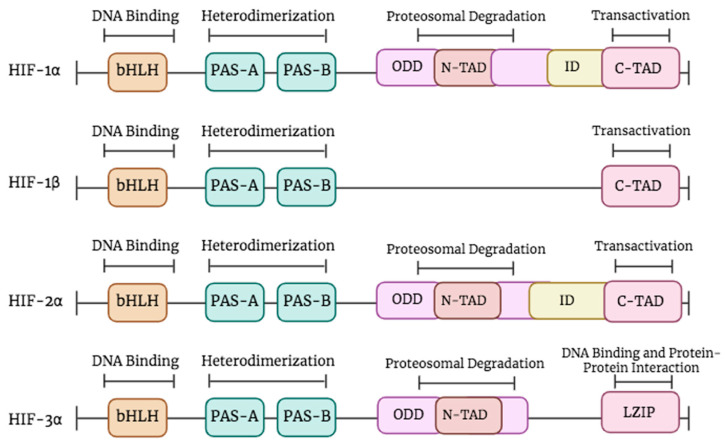
The structure of HIF proteins. The top line represents the functions of different domains. All HIF members contain a bHLH motif and two PAS domains (PAS-A and PAS-B). HIF-1β lacks the oxygen-dependent degradation (ODD), the N-TAD, and the inhibitory domain (ID). HIF-3α isoform exhibits an LZIP implicated in DNA binding and protein–protein interaction and lacks the C-TAD and ID domains. Created in BioRender.

The HIF gene family consists of three isoforms, namely HIF-1, HIF-2, and HIF-3 [11,12]. Every isoform is composed of a dimeric configuration comprising an alpha (α) subunit sensitive to oxygen and a beta (β) subunit insensitive to oxygen. β subunits are collectively referred as aryl hydrocarbon receptor nuclear translocator (ARNT) [12] (Figure 1).

HIF-1 and HIF-2 play crucial roles in the regulation of cellular oxygen homeostasis, although the precise function of HIF-3 remains less well defined. In the human body, there exist three distinct isoforms of the HIF-α subunit, namely HIF-1α, HIF-2α, and HIF-3α. Additionally, there are two isoforms of the HIF-β subunit, which are known as ARNT and ARNT2 [13,14]. In fact, the ARNT induces a wide range of genes involved in tumor growth and angiogenesis, which plays a major role in controlling carcinogenesis. ARNT was detected expressed in different types of cancers including breast, lung and prostate cancer [15].

The isoforms share certain components, such as the PAS-A and PAS-B sections that facilitate dimerization, and an HLH domain responsible for DNA binding [16] (Figure 1). Divergences manifest in the latter portion of the subunits, specifically HIF-1α and HIF-2α, which exhibit transactivation domains (TADs) known as N-TAD at the NH2-terminal and C-TAD at the COOH-terminal. In normoxic conditions, the transcriptional activity is inhibited by an inhibitory domain (ID) located between the N-TAD and the C-TAD. While in the case of hypoxia, the C-TAD interacts with co-activators in order to modulate the process of gene transcription [13,17].

The N-TAD and the oxygen-dependent degradation (ODD) region, which play a crucial role in maintaining protein stability, are exclusive to HIF-1α, HIF-2α, and HIF-3α [13]. Moreover, HIF-1β is deficient in both the N-TAD and ODD. The functional disparities between HIF-1α and HIF-1β can be attributed to their underlying structural contrasts. Specifically, transactivation domains in HIF-1α contribute to the augmentation of transcriptional activity, whereas HIF-1β predominantly serves as a partner for dimerization [13]. The presence of cellular oxygen determines the stability and activity of HIF isoforms. The degradation of HIF-1α in normoxia occurs via the ubiquitin–proteasome pathway, but HIF-1β remains stable and retains its functionality regardless of the presence of oxygen [18]. HIF-2α, the second constituent, exhibits structural characteristics similar to HIF-1α and governs iron metabolism and the expression of the erythropoietin (EPO) gene [19,20]. The regulation of angiogenesis and metabolic pathways is governed by the interaction between HIF-1α and HIF-1β [21]. HIF-3α, the third member, has sequence homology with HIF-1α and HIF-1β in both the bHLH-PAS and the ODD domain. HIF-3α can form heterodimers with HIF-1β. It has an N-TAD without the C-TAD domain. The precise functions of HIF-3α are still being investigated. Its interactions with HIF subunits, co-activators, and co-repressors form a complex gene regulatory network. This intricacy helps cells adapt to the specific demands of their microenvironment [9,13].

## 3. HIF-1α in Cancer: Oxygen-Dependent Regulation and Tumor Progression

**Figure 2:**
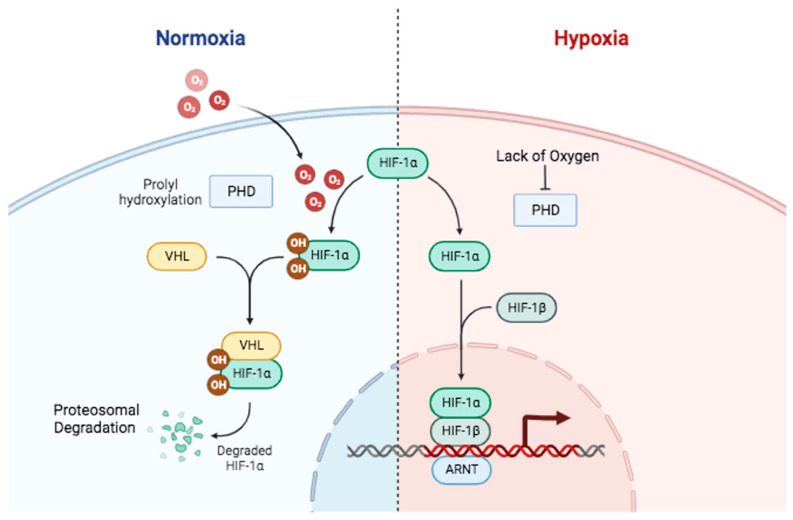
Oxygen-dependent mechanisms in regulating HIF-1 stability and function. In normal oxygen conditions (**left**), PHDs hydroxylate HIF-1α, stimulating the HIF-1α ubiquitination by E3 ligase VHL (Von Hippel–Lindau) and subsequently its proteosomal degradation. In low-oxygen conditions (**right**), PHDs and VHL are inhibited, preventing PHD hydroxylation activity. This allows HIF-1α to escape degradation and form a transcriptional complex that binds to specific regions of hypoxia response elements on the DNA of target genes, activating gene transcription. Created in BioRender.

Oxygen availability regulates HIF-1α expression in cells. Under normoxic conditions, HIF-1α is degraded rapidly with a half-life of approximately five minutes [22]. Its degradation is facilitated by a group of prolyl hydroxylase enzymes (PHDs) that are dependent on oxygen and iron [23]. PHD enzymes hydroxylate specific proline residues, Pro-402 and Pro-564, on HIF-1α in the presence of oxygen [5], which in turn facilitates the ubiquitination of HIF-1α by the Von Hippel–Lindau (VHL) E3 ligase enzyme, leading to its degradation by the ubiquitin–proteasome system (Figure 2) [23]. Additionally, factor-inhibiting HIF (FIH) hydroxylates an asparagine in the C-terminal transactivation domain of HIF-α, which inhibits the p300 co-activator and represses the transcriptional machinery [24].

In hypoxic conditions, PHD enzyme activity is impaired due to insufficient oxygen and the availability of cofactors such as iron and ascorbate [25]. Consequently, the hydroxylation of HIF-1α is inhibited, preventing its ubiquitination and subsequent degradation. In this scenario, the HIF-1α protein remains stable and accumulates in the cytosol. Therefore, HIF-1α relocates to the nucleus and binds with HIF-1β to form a functional heterodimer. This heterodimer regulates target gene transcription by recruiting p300/CBP co-activators and forming a functional HIF transcription complex (Figure 2). The complex binds to specific DNA sequences called hypoxia response elements found in the promoter regions of target genes [26]. This binding recruits co-activators and activates the transcription of target genes related to oxygen homeostasis. The target genes encompass those that encode proteins related to angiogenesis (e.g., Vascular Endothelial Growth Factor (VEGF)), glycolysis (e.g., GLUTs, glycolytic enzymes), tumor survival (e.g., Bcl-2), metastasis, and other factors facilitating adaptation to hypoxia [26].

HIF-1α stability and function can be regulated through non-hypoxic mechanisms. Signaling pathways, including growth factors, cytokines, and oncogenic signaling, can independently induce HIF-1α expression and activity regardless of oxygen levels. Downstream signaling cascades, such as PI3K/Akt/mTOR and Ras/MAPK pathways, are involved in enhancing HIF-1α synthesis or inhibiting its degradation [27,28].

The regulation of HIF-1α under varying oxygen levels requires a careful balance between its degradation through hydroxylation-dependent ubiquitin-mediated pathways and its stabilization and activation in response to hypoxia or non-hypoxic signals. This regulation enables cells to adapt to varying oxygen levels and maintain oxygen homeostasis.

HIF-1α plays a prominent role in tumorigenesis and cancer pathogenesis. Cancer often leads to an increased expression of this gene, which is influenced by both hypoxic and non-hypoxic mechanisms. This ultimately contributes to the advancement of cancer through interconnected pathways [29].

HIF-1α plays a crucial role in cancer by influencing cell division and proliferation. In cases of severe hypoxia, HIF-1α can cause cell cycle arrest by suppressing c-Myc and increasing p21 levels. p21 is a cyclin-dependent kinase inhibitor that functions as a checkpoint in the cell cycle. The role of HIF-1α in inducing cell cycle arrest during hypoxia varies depending on the specific context and tumor type being studied [30]. HIF-1α also plays a role in cell proliferation and survival. Hypoxia generally inhibits cell proliferation due to the increased oxygen demands associated with higher cell numbers, which further worsens hypoxic stress [31,32]. Interestingly, certain cell populations continue to proliferate even in hypoxic conditions. This persistence is consistent with the maintenance of stem cell populations found in low-oxygen environments and serves both normal and abnormal functions [33]. Studies indicate that hypoxic conditions can promote the activation of growth and survival signaling molecules, thereby facilitating the proliferation of cancer cells. These factors, which HIF regulates, include widely recognized molecules such as VEGF, Endothelin-1 (EDN1), Insulin-like Growth Factor-2 (IGF2), and Transforming Growth Factor-α (TGFA) [34].

Moreover, the participation of HIF-1α in the process of angiogenesis is of utmost importance. Hypoxia is a strong trigger for tumor angiogenesis, which involves the formation of a new network of blood vessels to provide necessary nutrients and oxygen to tumor cells. The activation of VEGF, a proangiogenic factor, by HIF is crucial for the initiation of angiogenesis in tumor cells, leading to accelerated tumor growth [35,36].

Metastasis is a characteristic feature of malignancy, wherein cancer cells possess the remarkable capacity to disseminate from the primary tumor to distant organs, giving rise to secondary tumors. HIF-1α facilitates epithelial-to-mesenchymal transitions (EMT) in this process. EMT is characterized by the downregulation of epithelial markers and the upregulation of mesenchymal markers, facilitating the detachment of cancer cells from neighboring cells and their migration to distant locations [37]. Research has shown that hypoxia can trigger EMT by regulating the expression of important EMT markers such as Twist and Snail transcriptional factors [38]. HIF-1α activates signaling pathways related to the TGF-β pathway and the PI3K/AKT/mTOR pathway, which are involved in EMT and metastasis [39].

Moreover, HIF-1α regulates the transcription of genes responsible for producing proteases that alter or break down the extracellular matrix, thereby promoting the invasion of cancer cells. These genes include PLAUR, MMP2, MMP9, MMP14, and CTSC, which encode for proteases such as plasminogen activator urokinase receptor (PLAUR), matrix metalloproteinases (MMP2, MMP9, MMP14), and cathepsin C (CTSC) [31]. HIF-1α is known to influence various factors that contribute to intravasation—the process by which cancer cells enter blood vessels. These factors include permeability factors such as VEGF and angiopoietin-2 (ANGPT2) as well as motility factors like autocrine motility factor (AMF) and mesenchymal–epithelial transition (MET) [40,41].

## 4. Metabolic Reprogramming in Hypoxia: Role of HIF-1α

Cancer cells undergo metabolic alterations and leverage a multitude of signaling pathways to sustain their growth, survival, and prolonged existence. Hypoxia emerges as a crucial factor in triggering metabolic reprogramming within tumors. This is achieved by elevating the requirements to produce essential metabolites, facilitating tumor growth, development, and gaining resistance to therapeutic interventions.

### 4.1. Hypoxia-Induced Glycolysis and Lactate Accumulation in Cancer Cells

**Figure 3:**
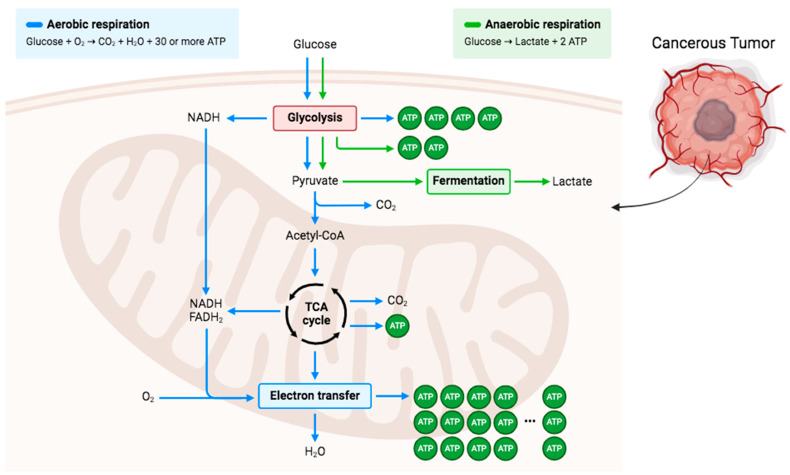
Overview of glucose metabolism in aerobic and anaerobic respiration. Glucose is the main energy source in the body in which it is converted to pyruvate to either be further converted and enter TCA cycle in aerobic conditions or be converted to lactate by fermentation in anaerobic conditions. Cancerous tumor cells demonstrated that the anaerobic respiration path tends to be followed regardless of whether there is enough oxygen present. Created in BioRender.

Cells derive energy in the form of ATP by oxidizing glucose, which is the primary macronutrient involved in cellular processes. Energy metabolism varies significantly between normal cells and cancer cells. Glycolysis is the cytoplasm’s primary cellular pathway for converting glucose into pyruvate. Under aerobic conditions, pyruvate is transported into the mitochondria and converted by the pyruvate dehydrogenase complex (PDH) to acetyl-CoA. Acetyl-CoA then enters the citric acid cycle, where it is further metabolized into carbon dioxide. Under anaerobic or hypoxic conditions, normal cells convert pyruvate produced from glycolysis into lactate, which is subsequently released from the cells (Figure 3). Cancer cells in low-oxygen conditions utilize anaerobic metabolism to produce lactate. During hypoxia, HIF-1α activates pyruvate dehydrogenase kinase 1 (PDK-1), leading to the phosphorylation and inhibition of PDH. Glycolysis induction and PDH inhibition promote lactate accumulation, which stabilizes HIF-1α, which is a highly active metabolic pathway in tumor cells. Despite sufficient oxygen availability, most cancer cells primarily rely on lactic acid fermentation and exhibit a high rate of glycolysis [26].

Physiological lactate levels in the bloodstream of healthy cells typically range from 1.5 to 3 mM. In the case of cancer cells, hypoxia leads to significant metabolic changes that result in a substantial increase in lactate concentration, ranging from 10 to 30 mM. This phenomenon leads to increased acidity within the intracellular environment of cancer cells [42]. Proton-like monocarboxylate transporters (MCTs) facilitate the movement of H+ and lactate to the extracellular space as a means of counteracting this process. Lactate efflux leads to acidification of the extracellular pH in the tumor microenvironment. The acidic pH is a characteristic of malignancy. These events hinder cell growth, but cancer cells can adapt, promoting tumor progression [43].

Cancer cells typically consume more glucose than normal cells. HIF-1α regulates several genes involved in glycolysis during hypoxia, including glucose transporters (GLUT1 and GLUT3), enolase 1, hexokinase 1 and 2, pyruvate kinase M2 (PKM2), phosphoglycerate kinase 1, and lactate dehydrogenase (LDHA) [44]. PKM2, a gene encoding pyruvate kinase M2, participates in various biological processes. Alternative splicing led to the production of two isoforms, namely PKM1 and PKM2 [45]. PKM2 expression is increased in cancer cells, promoting cell proliferation. The PKM2 enzyme functions as a co-activator for HIF-1α, facilitating the recruitment of co-activators, binding to chromatin, and inducing transcription [46,47]. Cancer cells undergo metabolic reprogramming and promote various cancer development mechanisms, such as angiogenesis [13].

HIF-1α promotes the conversion of glucose into glycogen during hypoxia to store sufficient energy for prolonged stress. Glycogen metabolism in cancer cells can be influenced by hypoxic conditions, leading to changes in both synthesis and breakdown rates. These changes are dependent on the specific metabolic needs of the cancer cells [13,48].

### 4.2. Hypoxia-Induced Alterations in Lipid Metabolism Pathways

Under hypoxic conditions, cells adapt by regulating the expression of genes associated with energy and lipid metabolism. Fatty acids (FAs) have important functions in energy production, membrane formation, triacylglycerol (TAG) storage, and signaling. They can be obtained from dietary intake or synthesized within the body. FAs are vital for the survival and proliferation of cells, including rapidly dividing tumor cells, in both normal and low-oxygen (hypoxic) conditions. This discussion explores the changes in lipid metabolism pathways caused by cancer [13].

**Figure 4:**
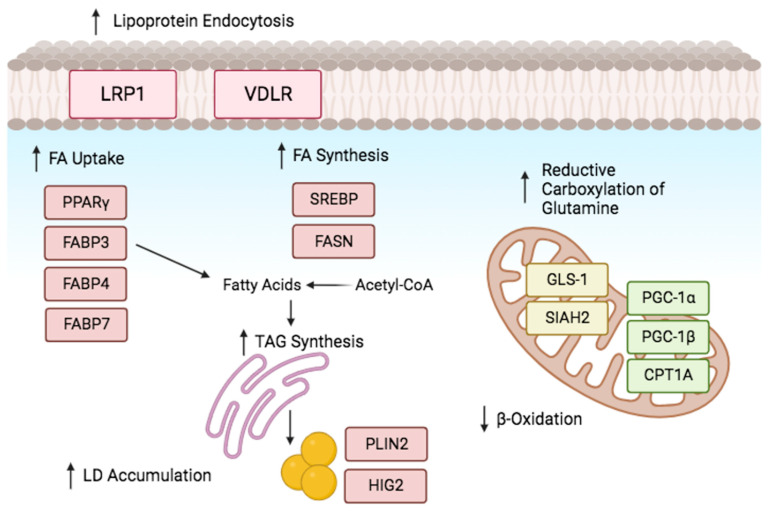
Lipid metabolism reprogramming under oxygen deprivation. Cells adjust to hypoxia by controlling the expression of genes related to lipid and energy metabolism. Hypoxia promotes lipogenesis by modulating the proteins involved in fatty acid (FA) uptake, production, storage, and use. Hypoxia promotes the uptake of extracellular FA by upregulating the production of FABPs and activating PPARγ. Upregulating VLDLR and LRP1 facilitates lipoprotein endocytosis. Reductive glutamine metabolism maintains citrate levels and acetyl-CoA production. SREBP-1 upregulates FASN expression, and fat accumulation is encouraged by β-oxidation inhibition. Created in BioRender.

HIF-1α plays a prominent role in low oxygen levels by directly activating the transcription factor gene peroxisome proliferator activated receptor (PPARγ) [49]. This activation increases in TAG synthesis and facilitates the uptake of FAs by cells [50]. Moreover, it was found that fatty acid binding proteins FABP3, FABP7, and FABP4 play a role in enhancing fatty acid uptake in cancer cells and primary mouse hepatocytes [51,52]. Figure 4 demonstrates that HIF-1α enhances lipoprotein endocytosis by increasing the expression of specific receptors, such as low-density lipoprotein receptor-related protein (LRP1) in vascular smooth muscle cells and very low-density lipoprotein receptor (VLDLR) in cardiomyocytes [53,54].

Furthermore, in conditions of hypoxia or impaired mitochondrial respiration, the reductive metabolism of glutamine plays a substantial role in providing carbon for the synthesis of fatty acids (Figure 4). This offers an alternative method to preserve de novo fatty acid synthesis during hypoxic conditions through the regulation of glutaminase 1 (GLS1), glutamate dehydrogenase 1 (GLUD1), and isocitrate dehydrogenase 1 (IDH1) by HIF-1α [55,56]. HIF-1α increases α-ketoglutarate production by upregulating GLS1 and E3 ubiquitin ligase SIAH2 [57], which subsequently controls the degradation of the α-ketoglutarate dehydrogenase complex (KGDH) [58].

Furthermore, the activation of sterol regulatory element-binding protein (SREBP-1), which upregulates the expression of fatty acid synthase (FASN), is facilitated by sufficient fatty acid supply through Akt and HIF-1α [59,60]. FASN, a multi-enzyme, plays a role in the initiating of fatty acid production [60]. The FASN enzyme complex plays a role in initiating fatty acid synthesis and has been linked to both chemoresistance induced by hypoxia and the progression of cancer [61].

Hypoxia inhibits fatty (FAs) acid breakdown, causing an increase in intracellular free fatty acids leading into lipotoxicity. Consequently, cells metabolize FAs by converting them into TAGs and storing them as lipid droplets (LDs). HIF-1α activates a phosphatidic acid phosphatase (Lipin-1) and acylglycerol-3-phosphate acyltransferase 2 (AGPAT2) enzymes, promoting LD accumulation in the TAG synthesis pathway [61,62].

AGPAT2, a direct target of HIF-1α, is elevated in cancer patient biopsies. For instance, increased expression of AGPAT2 was demonstrated in osteosarcoma, ovarian and gastric cancer [63]. Similarly, HIF-1α stimulates the expression of lipin-1, an enzyme involved in the synthesis of TAGs, through its role as a phosphatidic acid (PA) phosphatase (Figure 4) [61]. These enzymes play a crucial role in chemoresistance development and TAG accumulation in hypoxic conditions [64]. Moreover, PA and lysophosphatidic acid were highlighted as crucial precursors for phospholipids [65], which are essential for the formation of cell membranes. Hypoxia promotes the activation of essential constituents of membranes, leading to increased LD production.

HIF-1 induces HIG2/HILPDA (hypoxia-inducible protein 2/hypoxia-inducible lipid droplet associated) expression, encouraging lipid aggregation in both cancer and normal cells storage [66]. Moreover, hypoxia suppresses essential enzymes involved in fatty acid breakdown. HIF-1 and HIF-2 decrease the expression of carnitine palmitoyl transferase 1A (CPT1A), an enzyme responsible for transporting fatty acids into mitochondria, as well as proliferator-activated receptor-co-activator-1 (PGC-1), which is an important enzyme involved in fatty acid oxidation [49,67].

### 4.3. Amino Acid Metabolism

Numerous malignancies rely on amino acids, leading to a significant increase in their absorption and metabolism. Amino acids are of critical importance in facilitating the ability of cancer cells to tolerate various stimuli and facilitate their survival and proliferation, particularly in response to genotoxic, oxidative, and nutritional stressors. In hypoxia, the metabolic processes of non-essential amino acids, such as glutamine and serine, play a crucial role beyond their traditional role as essential components for protein synthesis [68,69].

Glutamine has been identified as a predominant fuel source for tumor cells, as it supplies carbon and nitrogen for various cellular processes including the tricarboxylic acid (TCA) cycle, biosynthesis, energy production, and maintenance of cellular homeostasis [58]. The constraints on pyruvate entrance into the TCA cycle caused by hypoxia result in an elevation of glutamine uptake due to the upregulation of glutamine transporters SNAT2/SLC38A2 and SLC1A5 [70]. Within the cellular environment, the enzyme glutaminase facilitates the conversion of the amino acid glutamine into glutamate [58]. This resulting glutamate molecule is then further metabolized into α-ketoglutarate (α-KG) through the action of either transaminase or glutamate dehydrogenase [58]. α-KG has the capability to undergo a process called reductive carboxylation, resulting in the production of isocitrate, citrate, or succinate [71]. These compounds are vital components of the TCA cycle. Under hypoxic conditions, the process of reductive carboxylation takes precedence over oxidative carboxylation, which is primarily facilitated by the activity of HIF-1α [58].

However, the E1 subunit of the α-ketoglutarate dehydrogenase complex undergoes ubiquitination and proteolysis, which are facilitated by the activation of HIF-1. The tumor suppressor gene Von Hippel–Lindau (VHL) has significant importance in this particular context, as mutations result in its inactivation. Consequently, this leads to an elevated accumulation of HIF-1 proteins and subsequent activation of transcription, including genes such as platelet-derived growth factor (PDGF) and VEGF. These events substantially impact on the physiology of cancer [72,73,74].

Moreover, the use of glutamine leads to the generation of aspartate, which is a crucial component for the synthesis of pyrimidines through de novo pathways. Moreover, glutamate produced from glutamine plays diverse biological roles, such as participating in the synthesis of glutathione, serving as a precursor for tumor growth, and contributing to the production of other amino acids [75].

Serine also plays a pivotal function in the formation of cancer cells. The proliferation of specific cancer cells has been observed to decrease when serine levels are depleted as demonstrated in both in vitro and animal models [76]. The multiple roles of serine include the generation of phospholipids, specifically phosphatidylserine, the biosynthesis of other amino acids such as cysteine and glycine and providing of one-carbon units within the folate pathway. In the presence of hypoxia, the biosynthesis of serine from glucose is facilitated by three enzymes, namely phosphoserine aminotransferase (PSAT1), phosphoserine phosphatase (PSPH), and phosphoglycerate dehydrogenase (PHGDH). PHGDH exhibits significant upregulation in various types of malignancies, including breast, cervical, non-small cell lung, and colorectal cancers [77]. HIF-1α induces the transcriptional activation of the cysteine transporter (SLC7A11) and the regulatory subunit of glutamate–cysteine ligase, known as GCLM, in order to enhance the synthesis of glutathione in specific cancer types [78].

## 5. Targeting HIF-1α as a Cancer Therapy

Hypoxia and its related HIF system were highlighted as possible cancer therapy approaches, since they are essential processes in cancer cells. In recent years, numerous approaches and medications have been investigated and used as therapeutic treatments to block HIF-1α activity and associated pathways. The fundamental strategy involves blocking the HIF-1α activation pathway’s several processes, including transcription, transcriptional activity, translation, stability, heterodimerization, transport into the nucleus, binding to DNA, and HIF target genes considering the entire HIF pathway processes. It is important to highlight that the inhibitors discussed in this publication are not exclusively required to demonstrate selectivity toward HIF-1. Instead, they contain the ability to inhibit other targets as well. The inhibitory activities were demonstrated on various cancer cell line models [79].

### 5.1. Inhibitors of HIF-1α mRNA Expression

**Figure 5:**
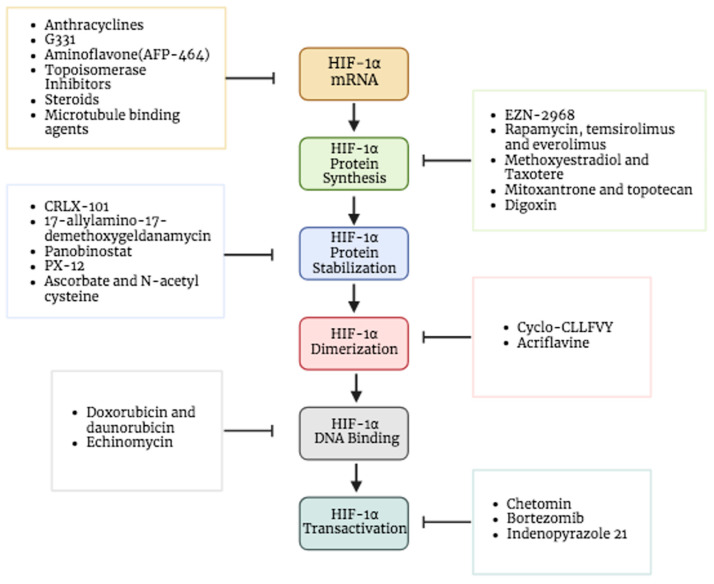
Targeting HIF-1α as cancer therapy. Different drugs used as HIF-1α inhibitors that target various HIF processes including HIF-1α mRNA expression, HIF-1α protein synthesis, HIF-1α protein stabilization, HIF-1α dimerization, HIF-1α DNA binding and HIF-1α transactivation. Created in BioRender.

The first step considered in the HIF pathway is the transcription of HIF-1α. Many agents were identified to prevent its transcription [80]. For instance, anthracyclines and aminoflavone are drugs that showed effects inhibiting the HIF-1α mRNA expression. AFP-464 is a prodrug that is for the time being in phase I clinical trials; it contains the active ingredient aminoflavone, which almost entirely blocks HIF-1 protein expression but slightly inhibits HIF-1α mRNA expression [81,82,83]. This suggests that aminoflavone reduces the translation of HIF-1α as well as its stability. In addition to topoisomerase inhibitors, steroids and microtubule-binding agents are other drugs that exhibit the inhibitory effect of the HIF-1α mRNA expression rate by various mechanisms [81] (Figure 5).

### 5.2. Inhibitors of HIFα Protein Synthesis

Some of the drugs that block the mRNA translation of HIF-1α involve (I) RNA interference drugs like EZN-2968, an oligonucleotide blocks the translation by binding to HIF-1α mRNA, (II) mTOR inhibitors like rapamycin, everolimus (RAD-001), and temsirolimus (CCI-779), (III) microtubule-targeting drugs like 2-methoxyestradiol and taxotere, (VI) COX-2 inhibitors like ibuprofen, (V) topoisomerase inhibitors like mitoxantrone and topotecan and (IV) cardiac glycosides like digoxin that has been employed to treat heart problems for years (Figure 5) [5,84,85]. Interestingly, the effect of digoxin as an HIF-1 inhibitor in human breast cancer tissues is under investigation through phase II clinical trials [86]. Moreover, Ganetespib, a novel and potent inhibitor of heat shock protein 90 (HSP90), has shown efficacy in suppressing HIF-1 as well [87]. This medicine has demonstrated promising efficacy in the treatment of various types of malignancies, including non-small cell lung cancer, breast cancer, and melanoma [88]. The ability of this medication to affect many pathways that are often altered in cancer makes it an intriguing candidate for overcoming resistance to other therapeutic methods [89]. Multiple clinical trials have evaluated the effectiveness of Ganetespib [90,91].

### 5.3. Inhibitors of Protein Stabilization and Accumulation

One of the targeted processes is the inhibition of HIF-1α protein stabilization and accumulation; many drugs assist in achieving this. For example, CRLX-101 is a nanoparticle drug designed to gradually release camptothecin (CPT) in tumors to accumulate into solid tumors (Figure 5). It showed an efficiency in inhibiting the protein accumulation of HIF-1α and hypoxic induction of VEGF mRNA in addition to protein expression. A number of tumor types are now being treated with CRLX101 in combination with other anticancer medications in phase II clinical trials [92].

On the other hand, another target for inhibition can be the stability of the HIF-1α protein. Certain inhibitors cause the HIF-1α protein to degrade, which reduces its stability. There are multiple drugs that can promote the degradation of its protein: HSP90 inhibitors (e.g., 17-allylamino-17-demethoxygeldanamycin), class II histone deacetylase (HDAC) inhibitors and the thioredoxin inhibitor (e.g., PX-12), which cause ubiquitination and the proteasomal degradation of HIF-1α. In particular, panobinostat is an inhibitor of histone deacetylase, which obstructs the HSP90/HDAC6 complex that interrelates with HIF-1α and hinders its degradation, hence preventing formation of the complex prompt degradation of HIF-1α [5,10,36,93]. Additionally, some antioxidants (e.g., ascorbate–vitamin C and N-acetyl cysteine) could be used to block tumor growth by inducing HIF-1α degradation. When a high level of ascorbate is present, PHD initiates the hydroxylation of HIF-1α, which prevents the binding of VHL and leads to proteasomal degradation [24,94]. Furthermore, it was also seen that ascorbate efficiently blocked HIF-1-dependent gene expression [94].

Other strategies could also be effective in protein degradation like a nucleic acid-based strategy by using G-rich oligonucleotides that target the HIF-1α protein rather than its mRNA. Also, some natural products such as berberine allow HIF-1α degradation induction and its anti-angiogenic effects in cancer cells and in endothelial cells [30,95,96,97].

### 5.4. Inhibitors of Dimerization

HIF-1 dimerization has been reported to be disrupted and its transcriptional activity inhibited by cyclic peptide inhibitor (cyclo-CLLFVY), which binds to the HIF-1α PAS-B region (Figure 5). HIF-1-mediated hypoxia response signaling decreased by Cyclo-CLLFVY in a variety of cancer cell lines without changing the operation of the HIF-2 isoform [98]. Likewise, acriflavine, an antibacterial drug that was clinically used before penicillin, was noticed to directly bind to the HIF-1α and HIF-2α PAS-B subdomain and prevents them from interacting with HIF-1β, preventing the transcription of genes controlled by HIF and impairing the development and vascularization of the tumor [99,100,101].

### 5.5. Inhibitors of DNA Binding

Some of the DNA-binding inhibitors are doxorubicin and daunorubicin, which were used as traditional chemotherapeutic drugs, and they exhibit cytotoxic activity through DNA intercalation. This substance penetrates the DNA structure and binds to the DNA, which causes DNA damage [102,103]. DNA intercalating drugs may be used to treat cancer by destroying cancer cells’ DNA and preventing them from proliferating. Additionally, these two medications successfully prevented HIF-1 from attaching to the target genes’ hypoxia response element sequences. In cultured cells, anthracyclines like doxorubicin and daunorubicin bind to DNA and inhibit HIF-1 and HIF-2 binding as well as the angiogenic growth factors expression, which interferes with tumor vascularization and growth (Figure 5) [104]. Echinomycin is another intercalating agent known by DNA binding in a sequence-specific manner [105,106]. This small molecule has been demonstrated to prevent HIF-1 from binding to the promoter of VEGF, specifically to the (5′-CGTG-3′) sequences on hypoxia response elements [107].

### 5.6. Inhibitors of Transcriptional Activity

Chetomin inhibits the binding of CBP/p300 with HIF-1 and HIF-2 by targeting the CH1 domain of this protein [108] (Figure 5). The FDA-approved proteasome inhibitor bortezomib targets the HIF-1 carboxyl-terminal transactivation domain, which interacts with the co-activator p300, to inhibit HIF-1 transcriptional activity [81]. However, treatment of the drug does not cause the interaction to be disrupted [109]. Furthermore, it was discovered that indenopyrazole, without any impact on HIF-1 protein synthesis or heterodimerization, significantly reduced the transcriptional activity of HIF-1 [81,110].

## 6. Employing Nanotechnology in HIF-1α-Targeted Cancer Therapy

Nanotechnology has been shown to be a promising approach in targeting HIF in cancer therapy [111,112,113,114,115]. It can have a role in regulating oxygen content, directly inhibiting HIF-1α expression or interfering with the HIF-1α signal to control tumor cells’ malignancy and associated resistance to therapy.

### 6.1. Nanocarriers of HIF-1α Inhibitors

Encapsulating HIF-1 inhibitors onto nanoparticles (NPs) was anticipated to result in better drug pharmacokinetics and HIF-1-a inhibiting combination cancer therapy. Cisplatin has been widely used in clinical treatments and laboratory studies. It is a typical platinum antineoplastic medication with obvious genetic damage. Drug resistance may be overcome by combining cisplatin and HIF inhibitors, such as a novel type of ACF-loaded cisplatin NPs coated by microporous silica gel [116]. In preclinical anti-tumor research, acridine acriflavine (ACF), a long-used anti-parasite and antibacterial antibiotic, is utilized to block HIF, EMT, p53 activation, and mitochondrial or endoplasmic reticulum autophagy. Lipid nanocapsules (LNCs) were utilized as carriers to lessen ACF’s toxicity. In numerous preclinical studies, acriflavine was identified as a strong HIF-1 inhibitor that decreased the expression of phosphorglycerate kinase 1 (PGK1), VEGF, and HIF-1α, which are HIF-1 target genes [81,99,117]. Synergy treatments can achieve dual effects, obtaining optimal therapeutic efficacy. Researchers proposed a nanosystem (ACF-MnO_2_) composed of MnO_2_ nanoparticles and ACF, which increased the content of oxygen in tumors, inhibited HIF-1 function, and improved RT efficiency by downregulating PD-L1. Additionally, yolk shell Cu2 xSe-PtSe (CSP) NPs functionalized with ACF drastically improved oxygen levels, hence inhibiting HIF-1α and resulting in RT efficiency [117,118]. Moreover, curcumin can be applied as a drug as ZnPc-Cur-S-OA NPs to combine with PDT for HIF-1 arrest and GSH exhaustion in B16F10 cells [116,119].

### 6.2. Gene Silencing Using siRNA and Nanoparticles in Cancer Therapy

The process of RNA interference (RNAi) mediated by short RNAs involves numerous steps [120]. When unknown genetic material, like viral DNA, attempts to enter, the host cells’ innate defense response known as RNAi is activated. When double-stranded oligonucleotides enter the cytoplasm, they encounter the ribonuclease DICER, which causes them to cleave into little (20 bp) nucleotide portions. These small RNA fragments are then divided into guide strands and passenger strands. When degrading the passenger strand, the RNA-induced silencing complex (RISC) subsequently attracts the guide strand, which is complementary to the desired mRNA. The Argonaute protein family catalyzes the mRNA strand degradation that occurs as a result of the RISC, which silences a particular gene [121].

Mutations in DNA or the overexpression of specific oncogenes can alter normal cellular processes and convert healthy cells into cancerous ones. However, post-transcriptional silencing can modify the expression of these genes causing cancer using carefully designed small interfering RNA (siRNA) [122].

It has been demonstrated that silencing HIF-1 gene expression is a promising strategy for targeted cancer treatment. Due to its sequence-based selectivity, siRNA, a potent gene therapy tool, has garnered much interest. The practical use of siRNA-based treatment is nonetheless constrained by several factors such as weak stability and specificity. Fortunately, nanoparticle-based smart drug delivery systems have proven outstanding performance in overcoming such challenges and enhancing the therapeutic effects of RNA medicines. The advantageous features of nanoparticles include their ability to be customized in terms of form and size, biological behavior, capabilities to shield siRNA from destruction, and encouragement of the transport of siRNA through biological obstacles [123].

HIF-1α siRNA cancer therapy can utilize delivery systems based on whether the tumors are oxygen-deprived or not. Targeted delivery systems can be employed depending on HIF-1α expression levels in these hypoxic or non-hypoxic tumors. High effectiveness of the transfection can be seen in cationic liposomes coated with foliate that contain HIF-1α siRNA. Other than lipids, polymers can also be utilized to encapsulate HIF-1α siRNA, including D-tocopheryl polyethylene glycol 1000 succinate (TPGS), chitosan, carbon-based nanoparticles, as well as polyethyleneimine (PEI) [124].

Tumor resistance to a variety of treatment methods is boosted by hypoxia and HIF-1. As a result, combining HIF-1 siRNA-mediated gene therapy with other forms of treatment might greatly enhance the efficacy of treatment of cancer. HIF-1 gene silencing improved tumor cell susceptibility in cancer cell lines to chemotherapy agents including doxorubicin [125], cisplatin [126], gemcitabine [127], and 5-fluorouracil (5-FU) [128], and it also reduced the MDR1 gene and its product, which is P-glycoprotein (P-gp). P-gp is a protein that is produced by the MDR1 gene, and it was first identified in cancer cells. In order to load both macro-molecules with micro-molecule medications for chemotherapy and gene therapy, external carriers such as liposomes and micelles are required [124].

The use of nanoparticles for gene silencing of HIF-1 is still an active area of research, and various nanoparticle formulations and delivery strategies are being explored. However, further studies and clinical trials are necessary to optimize the nanoparticle design, assess their safety, and evaluate their therapeutic efficacy in cancer disease.

### 6.3. Oxygen Nano-Modulator

As previously explained, the degradation of HIF-1α begins with its necessary marking for degradation. This marking is accomplished by hydroxylating the proline amino acid residue within HIF-1α. This process occurs under normoxic conditions, which are characterized by adequate levels of molecular oxygen (O_2_). This mechanistic explanation offers valuable insights into the degradation pathways and reveals alternative approaches for the targeted therapeutic intervention of HIF-1α in cancer. Nanotechnology shows promise in the modulation of HIF-1α degradation through the use of nanomaterials and precise regulation of oxygen supply, as discussed in the literature [129].

Research investigating this concept has employed various methodologies, primarily focusing on different oxygen carriers, particularly hemoglobin-based carriers. Hemoglobin plays a crucial role in adapting to changing physiological conditions in tissues and arteries due to its ability to bind and release oxygen. When present in the circulation, the unbound form of hemoglobin is unable to effectively donate oxygen due to its instability and the weak blood flow.

Thus, hemoglobin-based oxygen carriers such as polyethylene glycol-conjugated hemoglobin (PEG-Hb) and glutaraldehyde-polymerized hemoglobin have been employed. This has been applied by researchers who reported how the polyethylene glycol-conjugated hemoglobin (PEG-Hb) treatment prevented HIF-1 from increasing and hence reducing the VEGF expression, which is the downstream protein that is strongly linked to tumor angiogenesis. Hence, it could be a novel anti-angiogenesis cancer treatment [130]. Moreover, these researchers continued and looked into the enhanced use of Hb-loaded nanoliposomes following oxygen saturation to transfer oxygen molecules into tumors in order to significantly reduce VEGF and improve the hypoxic microenvironment [131].

Other researchers focused their attention on EMT, which is a different biological mechanism that occurs during tumor spread. To minimize metastasis and impede tumor growth, they paired hemoglobin–liposome with various chemotherapeutic drugs, such as cabazitaxel [CBZ], 5-fluorouracil, and cisplatin. Both Western blot and immunostaining showed that hemoglobin–liposomes were effective at reducing tumor invasiveness by lowering levels of E-cadherin, MMP-2, or the EMT marker vimentin [132].

Despite these hemoglobin-based oxygen carriers (HBOCs) having a high oxygen carrying capacity, their stability is poor, and most of them are only useful for intravascular imaging. A fresh approach to addressing these issues is provided by the synthesis of perfluorocarbon (PFC) NPs and ultrasonic stimulation of oxygen transport [133]. Based on this, PFC-loaded NPs were developed as ultrasonic contrast agents for the regulation of anoxic malignancies by oxygen transport. The phase-deformable nano-agent (PFC-PLLA) that was created was made of a liquid PFC core and a methoxy poly (ethylene glycol)-b-poly(L-lactide) shell that effectively released oxygen when stimulated by ultrasound [133]. This prevented the build-up of HIF-1 and its downstream cytokines, including VEGF and glucose metabolic factors (HK2 and GLUT1), while also having an impact on glycolysis [133]. As a result, the use of bifunctional PFC-PLLA nano-agents in conjunction with ultrasonic imaging enhances both the efficiency of the latter and its ability to deliver oxygen [134].

## 7. Challenges and Limitations and Future Perspectives

Several approaches and medications have been investigated and suggested as therapeutic methods to block HIF activity and associated pathways in recent years, although there may be issues and concerns. For instance, as a consequence of tolerance restrictions, a lack of hypoxia specificity, or the selectivity on HIF isoforms, only a small number of them are now undergoing clinical trials.

The most prevalent types of cancer are solid tumors, which cause significant levels of mortality and morbidity worldwide [135]. Tumors that are solid in origin frequently exhibit hypoxic microenvironments, leading to cancer treatments like conventional chemotherapy, radiation, and immunotherapy as well as recently created molecular targeted therapy, which is less effective in treating cancer. Aggressive cancer behavior, a poor prognosis and more malignant phenotypes are all caused by the active HIF pathway, which is primarily regulated by HIF-1 and HIF-2 subunits.

Since the early 1990s, HIFs have been used as targets for the creation of new cancer therapies [136]. Many HIF inhibitors were recently created, and a few of them are now being tested in clinical studies. Some types of inhibitors directly affect HIF-1 or HIF-2 mRNA or protein level, α subunit and β subunits dimerization, or the interaction of HIF and the co-activators. However, the majority of them have various actions or operate as indirect inhibitors. Therefore, creating more specialized HIF inhibitors remains a challenging task. Solid tumors have been treated using HIF inhibitors in several clinical trials. Unluckily, due to safety concerns or poor therapeutic effectiveness, no medicines that directly block HIFs have yet been licensed for the treatment of cancer patients. HIF-1 and HIF-2 regulate how cells adapt to hypoxia and demonstrate a high sequence identity level. Although HIF-1 and HIF-2 are important in the advancement of cancer, HIF-1α dominates the acute hypoxia response, and the response to persistent hypoxia is driven by HIF-2 [137]. Of particular significance, HIF-2α expression is increased when one HIF-1α subunit is knocked down due to a reciprocal regulatory mechanism. A process for more combativeness tumor growth and medication resistance is provided by this shift from HIF-1 to HIF-2-dependent methods [13]. Interestingly, these studies have given a hint that in order to decrease hypoxia-inducible pathways, it may be necessary to target HIF-1α and HIF-2α simultaneously instead of just one component. However, the bulk of HIF inhibitors on the market now focus on HIF-1α. Therefore, creating HIF-2α inhibitors and drugs that may concurrently block HIF-1α and HIF-2α may improve more potent cancer treatment techniques.

The failure of clinical studies is also a result of poor patient selection. Regardless of the tumors’ levels of HIF expression, advanced or resistant solid tumors in patients are often enrolled in clinical studies to test the effectiveness of HIF inhibitors. In this situation, patients with low levels of HIF expression may not experience the therapeutic benefits of HIF inhibitors. In order to make HIF inhibitor clinical trials more successful for a particular subset of patients whose tumors have increased HIF levels, personalized medicine should be included in their design.

## 8. Conclusions

The significance of hypoxia-inducible factor-1 (HIF-1) in the advancement of cancer has been emphasized in this review along with its potential as a useful target for oncotherapy. The complexity of HIF-1’s regulation mechanisms and its extensive influence on crucial cancer processes like as metabolism, metastasis, and angiogenesis offer potential obstacles as well as opportunities for therapeutic intervention. Novel approaches, such as genetic silencing and small chemical inhibitors, appear to have potential in preventing cancers from adapting to hypoxic conditions. These strategies can address the issues of tumor heterogeneity and resistance, especially when paired with other targeted medicines. Future research is necessary to enhance the effectiveness and safety of these treatments, comprehend resistance pathways, and proficiently include them into established therapy frameworks. Research on HIF-1 targeting in cancer therapy is an important and developing field that has the potential to significantly alter current treatment modalities. It is essential to keep researching and disrupting HIF-1’s carcinogenic pathways to advance cancer treatment and improve patient outcomes.

## Data Availability

The raw data supporting the conclusions of this article will be made available by the authors on request.
